# Host soluble plasma factors increase dual-species *Staphylococcus epidermidis* and *Candida albicans* biofilm biomass without enhancing stress tolerance

**DOI:** 10.1038/s41598-026-49557-1

**Published:** 2026-04-22

**Authors:** Pavlína Vávrová, Ondřej Janďourek, Débora Cristina Coraça-Huber, Christopher Spiegel, Petr Nachtigal, Martin Krátký, Klára Konečná

**Affiliations:** 1 Faculty of Pharmacy in Hradec Králové, Department of Biological and Medical Sciences, Charles University, Akademika Heyrovského 1203/8, 500 03 Hradec Králové, Czech Republic; 2 Research Laboratory for Biofilms and Implant Associated Infections (BIOFILM LAB), University Hospital for Orthopaedics and Traumatology, Medical University of Innsbruck, Müllerstraße 44, 6020 Innsbruck, Austria; 3 Faculty of Pharmacy in Hradec Králové, Department of Organic and Bioorganic Chemistry, Charles University, Akademika Heyrovského 1203/8, 500 03 Hradec Králové, Czech Republic

**Keywords:** *Staphylococcus epidermidis*, *Candida albicans*, Dual-species biofilms, Host-biofilm interaction, Human plasma, Antimicrobial tolerance, Biotechnology, Microbiology

## Abstract

*Staphylococcus epidermidis* (*S. epidermidis*) and *Candida albicans* (*C. albicans*) are common members of the human microbiome and opportunistic pathogens, forming mixed biofilms, leading to catheter-associated bloodstream infections or wound infections. They are increasingly difficult to treat, highlighting the urgent need for new antibiofilm strategies. Understanding how host environmental factors affect microbial communities is crucial for their development. Based on our previous findings, we investigated whether higher levels of host soluble factors in human plasma (HP) and freeze-thaw lysed sheep red blood cells (FT-RBC) support the formation of *S. epidermidis*-*C. albicans* dual-species biofilm and increase resilience. Tryptic soy broth, RPMI 1640, and Lubbock media with HP or FT-RBC supplementation were used for in vitro biofilm formation. Total biomass, individual microorganisms, key matrix components (carbohydrates, proteins, eDNA), and antimicrobial tolerance were evaluated. Our results showed that although higher concentrations of HP in Lubbock medium support the formation of complex dual-species biofilm biomass, this does not correlate with enhanced antimicrobial tolerance. In contrast, higher adaptive resistance was detected in less heterogeneous biofilms formed under nitrogen-limited conditions in RPMI 1640-supplemented medium. These findings indicate that biofilm resilience is not solely dependent on biomass amount and complexity, underscoring the need to better understand host-biofilm interactions.

**Supplementary Information:**

The online version contains supplementary material available at 10.1038/s41598-026-49557-1.

## Introduction

*Staphylococcus epidermidis* (*S. epidermidis*) and *C. albicans* (*C. albicans*) are common human commensals that asymptomatically colonise multiple body sites, including skin and mucosal surfaces, where their proliferation is typically regulated by host immune defences^1,2^. However, if the host immune system is compromised, these microorganisms assume a pathogenic role, leading to infection^3,4^. Of particular concern is the global dissemination of multidrug-resistant lineages of *S. epidermidis*, while multidrug resistance is also strongly associated with *C. albicans*^5,6^. Another significant problem is the ability of these microorganisms to adhere to catheters and various indwelling medical implants, to form mixed biofilms, and to cause life-threatening bloodstream infections. *S. epidermidis* is the first common co-isolated microorganism with *C. albicans*, emphasising the growing threat of polymicrobial inter-kingdom infections in medical settings^3^. Not only coexistence, but also mutual beneficial cooperation of these two microbial agents was revealed. *C. albicans* facilitates the strong adhesion of *S. epidermidis* by providing a 3D scaffold through its hyphal structures^7,8^. This interaction is further enabled by key fungal surface components, namely Als proteins and *O*-mannosylations^7^. These abilities are a crucial mechanism for bacterial attachment and polymicrobial biofilm maturation^7,8^. Moreover, cooperation in dual-species biofilm communities composed of *S. epidermidis and C. albicans* has been shown to enhance virulence and reduce susceptibility to antimicrobial agents^9^.

The enhanced antimicrobial tolerance observed in biofilm-associated communities is multifactorial. A principal determinant is the extracellular polymeric matrix, which acts as a physical and chemical barrier, significantly impeding the diffusion of antimicrobial agents. Additionally, the inherent structural complexity and spatial heterogeneity of the biofilm architecture create distinct microenvironments. Within these niches, microorganisms are subjected to nutrient and oxygen limitations, prompting physiological adaptations such as metabolic downregulation and entry into a dormant or persister state. Persistent cells display restricted synthesis of macromolecules and arrested growth. Since many antimicrobials target cells that are actively growing and replicating, the presence of persisters may interfere with antimicrobial action, enabling these cells to tolerate a wide range of antimicrobials^10^. These mechanisms are classified as adaptive strategies that, together with acquired resistance mechanisms, increase the overall recalcitrance of the polymicrobial community to antimicrobial agents^10,11^.

The escalation of resilience within biofilm communities has significant clinical implications, contributing to increased mortality, prolonged hospitalisation, higher treatment costs, and frequent failure of standard therapies^12^. These challenges underscore the urgent need for new, effective antibiofilm approaches. An essential first step in developing such interventions is their evaluation against biofilms formed in vitro^13^.

However, the ability of microorganisms to form biofilms is profoundly influenced by environmental conditions, including nutrient availability and host-derived molecular signals. To ensure accurate and clinically relevant predictions, it is critical to consider the specific nutritional conditions of the host environment where the infection originates.

These host-derived soluble factors, such as iron, nitric oxide, glucose, and other plasma components, act as environmental signals that influence microbial growth rates and modulate gene expression associated with adhesion, matrix production, and virulence, including overall biofilm formation^14–19^. Host soluble factors in HP facilitate *S. epidermidis* adhesion to host tissues or implanted medical devices, and influence *S. epidermidis* gene expression, which further enhances biofilm formation^20^. Similarly, HP promotes *C. albicans* adhesion to implanted medical devices, upregulates *C. albicans* virulence gene expression, and induces the yeast-to-hyphal transition, contributing to robust biofilm formation with three-dimensional, multilayered architecture^21–23^. The interplay between these nutritional and host-related factors shapes microbial adaptation strategies and plays a crucial role in determining the structural and functional characteristics of biofilms formed in vivo^14^. A deeper understanding of these interactions seems essential for designing experimental conditions reflecting the host environment for the successful development of targeted anti-biofilm therapies.

In our previous study^24^, with methicillin-resistant *Staphylococcus aureus* (MRSA) and *C. albicans*, it was revealed that the most robust and heterogeneous dual-species MRSA-*C. albicans* biofilm with the highest tolerance to selected antimicrobials was formed in the medium with the highest amount of host soluble factors provided by HP – the Lubbock medium.

Lubbock medium is widely employed in biofilm research because it closely mimics the nutrient composition and physiological conditions encountered by microorganisms in vivo, particularly within host wound environments. This medium is also used for the cultivation of polyspecies communities and for investigating biofilm development and antibiofilm strategies^25–27^.

Although MRSA and *S. epidermidis* belong to the same genus, there is a fundamental difference in the virulence of these two microorganisms. MRSA is a highly virulent pathogen whose pathogenicity is driven by a wide range of toxins, immune-evasion proteins, and tissue-invasive enzymes. These include hemolysins, TSST-1, PVL, Protein A, and coagulase^28,29^. In contrast, *S. epidermidis* lacks these classic toxins and instead relies almost entirely on its ability to form robust biofilms, primarily through the production of polysaccharide intercellular adhesin (PIA) encoded by the *ica* operon^30^.

Given these fundamental differences in virulence strategies between MRSA and *S. epidermidis* and considering our previous findings demonstrating the influence of host-derived soluble factors on MRSA-*C. albicans* dual-species biofilm formation, we aimed to evaluate how an increased presence of host soluble factors in the cultivation medium supports the development of *S. epidermidis*-*C. albicans* dual-species biofilms *in vitro.*

Specifically, we focused on the impact of media, supplemented with host soluble factors present in human plasma (HP) and additionally freeze-thaw-lysed sheep red blood cells (FT-RBC). Among these factors belong primarily proteins such as albumin, transferrin, and components of the complement system, as well as the haemoglobin derived from FT-RBC. In general, host proteins can act as environmental cues, signalling to microorganisms that they are in a host environment. These host-derived factors can influence biofilm development by altering microbial attachment, modulating matrix production, and shaping the structure and stability of the biofilm, which in turn can contribute to either increased or decreased tolerance to antimicrobial agents^19,31^.

Thus, in our study, particular attention was paid to *S. epidermidis*-*C. albicans* dual-species biofilm robustness and complex architecture, the quantity of essential macromolecules within the biofilm matrix, and the most importantly, the ability to withstand environmental stress induced by the combined action of selected antimicrobial drugs.

## Results

### Focus on different nutrient availability in cultivation media for in vitro formation of *S. epidermidis* and *C. albicans* mono-species and *S. epidermidis*-*C. albicans* dual-species biofilms

Three cultivation media with different nutrient availability were chosen for biofilm formation in vitro: Tryptic soy broth (TSB), RPMI 1640 with L-glutamine, sodium bicarbonate, and glucose (RPMI), and the Bolton medium. To simulate some relevant host soluble factors, all media were supplemented with HP. TSB and RPMI were supplemented with 10% (v/v) HP. The Bolton medium was supplemented with 50% (v/v) HP and 5% (v/v) FT-RBC for the preparation of the Lubbock medium with slight modification^25^.

The level of available key macronutrients supply for dual-species biofilm formation is presented in Table S1 (Supplementary information). As shown in this table, the Lubbock medium possesses the highest nitrogen content. Moreover, this medium has the lowest glucose content.

### The impact of available nutrients on the total biomass of *S. epidermidis* and *C. albicans* mono-species and the *S. epidermidis*-*C. albicans* dual-species biofilms

The crystal violet (CV) staining method described by Christensen et al.^32^. was employed for the quantitative analysis of the *S. epidermidis* and *C. albicans* mono- and *S. epidermidis*-*C. albicans* dual-species total biofilm biomass production after 24 h of biofilm formation. The categorisation of biofilm producer phenotypes for both *S. epidermidis* and *C. albicans* strains alone, as well as for the *S. epidermidis*-*C. albicans* dual-species consortia, was done according to criteria introduced by Stepanović et al.^33^.

As is evident from Table 1, S. *epidermidis* was not able to form mono-species biofilm communities sufficiently in both RPMI + HP and the Lubbock media to be categorised as a biofilm producer. According to biofilm biomass production in the TSB + HP medium, this strain can be categorised as a weak biofilm producer.

**Table 1:** Categorization of the biofilm-producing phenotype of *S. epidermidis*, *C. albicans* mono-species, and *S. epidermidis*-*C. albicans* dual-species consortia.

Microorganism(s) forming biofilm consortia	Cultivation medium		
	TSB + HP	RPMI + HP	Lubbock
*S. epidermidis*	+	-	-
*C. albicans*	+	+	++
*S. epidermidis*-*C. albicans*	++	++	++
No biofilm producer(s)			-
Weak biofilm producer(s)			+
Moderate biofilm producer(s)			++

*S. epidermidis* (ATCC 35983) mono-species biofilm consortia, *C. albicans* (ATCC 90028) mono-species biofilm consortia, and *S. epidermidis*-*C. albicans* dual-species biofilm consortia were cultivated in vitro in different cultivation media for 24 h. TSB + HP – Tryptic soy broth supplemented with 10% (v/v) human plasma (HP), RPMI + HP – RPMI 1640 medium supplemented with 10% (v/v) HP, Lubbock – Bolton broth medium supplemented with 50% (v/v) HP, and 5% (v/v) freeze-thaw lysed sheep red blood cells. For the categorization of the biofilm-producing phenotype, the crystal violet staining method was employed.

The highest amount of *C. albicans* mono-species biofilm biomass was registered after biofilm formation in the Lubbock medium, which is the medium with the highest supplementation of HP. Therefore, it can be deduced that a higher proportion of HP does not adversely affect the formation of *C. albicans* mono-species biofilms.

Based on the semi-quantitative evaluation of *S. epidermidis*-*C. albicans* dual-species biofilm biomasses, it can be concluded that the mutual interaction of *S. epidermidis* and *C. albicans* represents a beneficial inter-kingdom partnership. The level of *S. epidermidis*-*C. albicans* dual-species biofilms allowed for categorisation of the participants as moderate biofilm producers, which corresponds to the same or one category higher classification, compared to *S. epidermidis* and *C. albicans* mono-species biofilms. After closer examination, the largest biomass of *S. epidermidis*-*C. albicans* dual-species consortia, composed of both microbial cells and biofilm matrix, was achieved in Lubbock medium, while the lowest was observed in the TSB + HP medium (see Fig. S1 in Supplementary information).

### Mutual interaction of *S. epidermidis* and *C. albicans* leads to the formation of a higher amount of *S. epidermidis*-*C. albicans* dual-species total biofilm biomass in comparison to mono-species biofilms

The same CV staining method mentioned above was employed to investigate how interactions between two microbial agents, *S. epidermidis* and *C. albicans*, influence the formation of biofilm communities. Both mono-species and dual-species biofilms were established to assess the cooperative interaction between *S. epidermidis* and *C. albicans* in 24-hour-old dual-species biofilms.

**Fig. 1:**
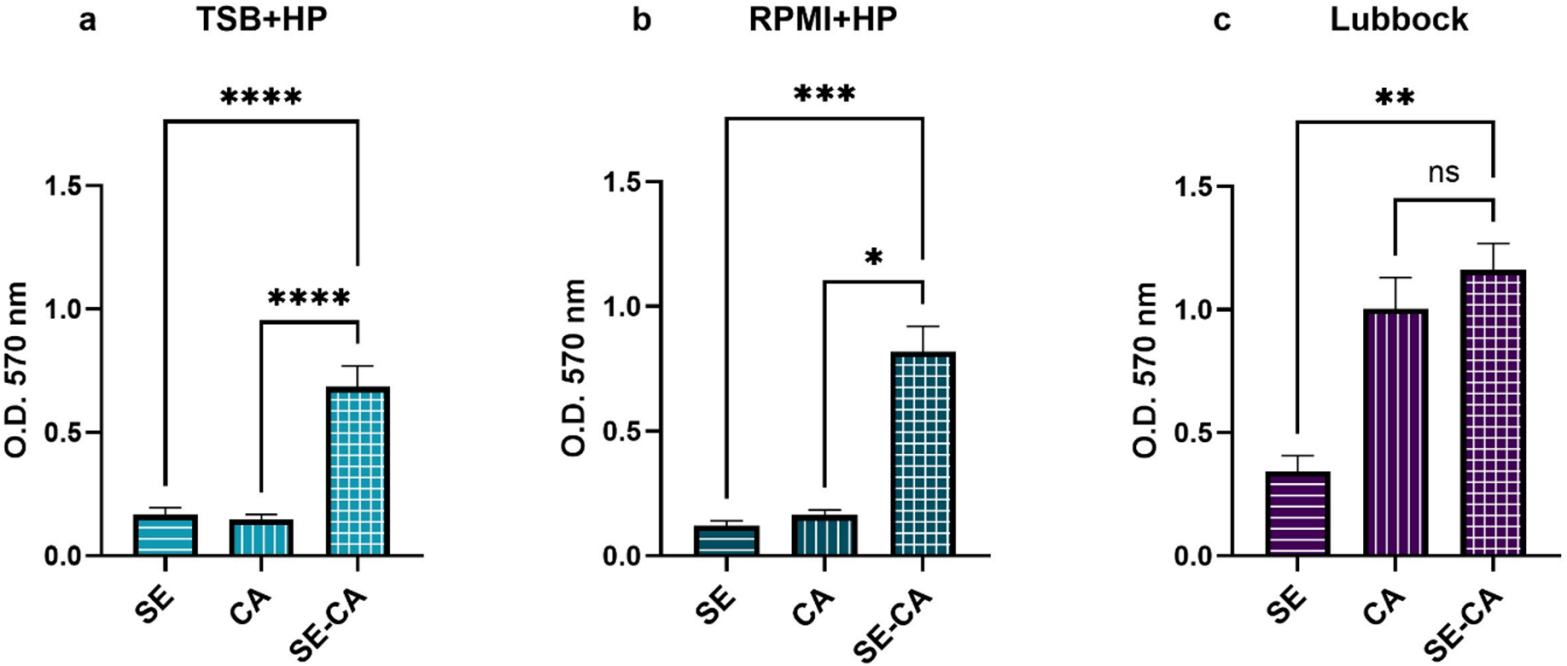
Impact of *S. epidermidis* and *C. albicans* interaction on total biofilm biomass formed under different nutritional conditions. The crystal violet staining method was employed for quantification of 24-hour-old total biomasses of *S. epidermidis* (SE, ATCC 35983), *C. albicans* (CA, ATCC 90028) mono-species, and *S. epidermidis*-*C. albicans* dual-species biofilm consortia (SE-CA). The values represent the mean ± SEM. Values from the negative control (individual cultivation medium without microorganisms) were subtracted, and the resulting data were analysed using one-way ANOVA or Kruskal-Wallis test, and a *p*-value < 0.05 was accepted as statistically significant. **a** TSB + HP – Tryptic soy broth supplemented with 10% (v/v) human plasma (HP): SE vs. SE-CA: *p* < 0.0001; CA vs. SE-CA: *p* < 0.0001; **b** RPMI + HP – RPMI 1640 supplemented with 10% (v/v) HP: SE vs. SE-CA: *p* = 0.0007; CA vs. SE-CA: *p* = 0.0152; **c** Lubbock – Bolton broth supplemented with 50% (v/v) HP and 5% (v/v) freeze-thaw lysed sheep red blood cells: SE vs. SE-CA: *p* = 0.0012; CA vs. SE-CA: *p* = 0.8320. *O.D.* optical density, *ns* not significant.

As shown in Fig. 1, mutually beneficial cooperative interactions between *S. epidermidis* and *C. albicans*, resulting in statistically significant differences in *S. epidermidis*-*C. albicans* dual-species biofilm biomass production compared to mono-species biofilms, were recognised after cultivation in both TSB + HP and RPMI + HP media. However, conditions provided by the Lubbock medium did not exhibit any significant benefit for *C. albicans* resulting from the mutual interaction with *S. epidermidis*.

### High *S. epidermidis*-*C. albicans* dual-species biomass production in the Lubbock medium is not related to the prosperity of individual participants in terms of their proliferation potential

Following the previous investigations of how *S. epidermidis* and *C. albicans* interactions influence biofilm formation, attention was focused on the abundance of *S. epidermidis* and *C. albicans* cells in dual-species communities. The ability of microorganisms to prosper in biofilm communities formed in different cultivation media, with the involvement of additional microbial participants, can be quantified using the multiplication ratio. To calculate the multiplication ratios, colony-forming units (CFU) were quantified using the spread plate technique. Multiplication ratios were calculated as the ratio between the CFU/mL present in the biofilm consortia after 24 h of cultivation and the CFU/mL present at the beginning of cultivation. For statistical analysis and better visualization, the ratios were log-transformed and are presented in the graphs as log (multiplication ratio).

**Fig. 2:**
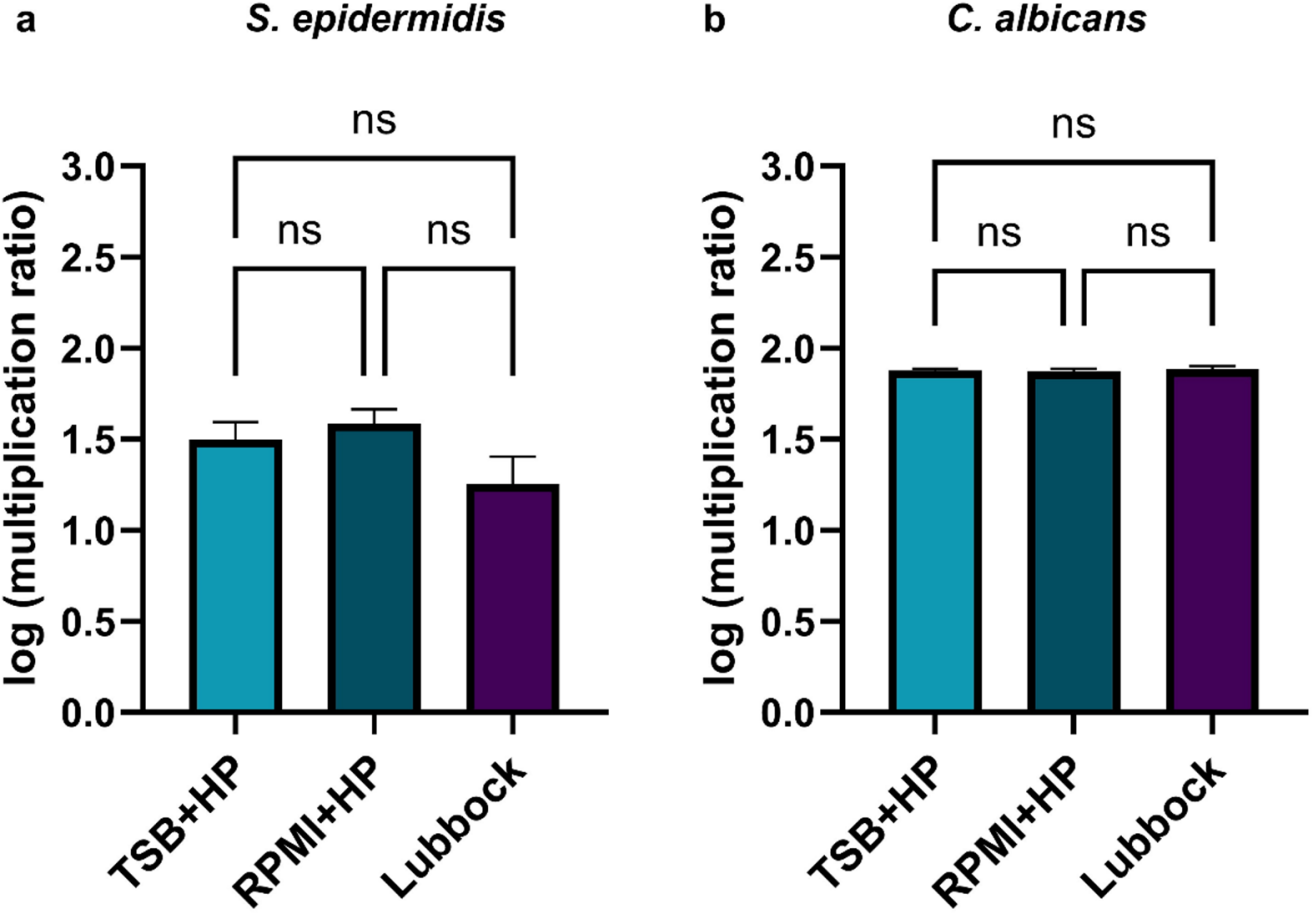
Proliferation of biofilm-forming agents in *S. epidermidis*-*C. albicans* dual-species consortia formed under different nutritional conditions. The log-transformed multiplication ratio represents the ratio between the number of colony-forming units/mL (CFU/mL) in dual-species *S. epidermidis* (ATCC 35983) and *C. albicans* (ATCC 90028) biofilms corresponding to individual microbial species after 24-hour cultivation and the CFU/mL of the initial inoculum. TSB + HP – Tryptic soy broth supplemented with 10% (v/v) human plasma (HP); RPMI + HP – RPMI 1640 supplemented with 10% (v/v) HP; Lubbock – Bolton broth supplemented with 50% (v/v) HP and 5% (v/v) freeze-thaw lysed sheep red blood cells, ns – not significant. The data represent the mean ± SEM. The data were analysed using one-way ANOVA or Kruskal-Wallis test, and a *p*-value < 0.05 was accepted as statistically significant. (a) TSB + HP vs. RPMI + HP: *p* = 0.8727; TSB + HP vs. Lubbock: *p* = 0.3126; RPMI + HP vs. Lubbock: *p* = 0.1772. (b) TSB + HP vs. RPMI + HP: *p* > 0.9999; TSB + HP vs. Lubbock: *p* > 0.9999; RPMI + HP vs. Lubbock: *p* > 0.9999.

As shown in Fig. 2a, in the case of *S. epidermidis*, no statistically significant differences in the multiplication ratio of *S. epidermidis* in *S. epidermidis*-*C. albicans* dual-species consortia were observed for the respective selected cultivation media. As illustrated in Fig. 2b, *C.* *albicans* in the community with *S. epidermidis* can prosper equally in all chosen media. Therefore, in the context of these findings, it can be assumed that the recognised statistically significant difference in the total biofilm biomass for *S. epidermidis*-*C. albicans* dual-species biofilms formed in the Lubbock medium may be conditioned by a larger amount of the biofilm matrix. In addition, the relative species-specific abundance was calculated and is summarised in Table S2 (Supplementary information).

### Higher concentration of host soluble factors derived from human plasma does not increase the tolerance of *S. epidermidis*-*C. albicans* dual-species biofilms to selected combined antimicrobial drugs

To explore a potential correlation between the concentration of host soluble factors, the amount of biofilm biomass, and the ability of biofilm communities to tolerate stress induced by the combined action of antimicrobial agents, experiments were conducted to assess the metabolic activity of biofilm-forming participants after exposure to selected antimicrobial agents. *S. epidermidis*-*C. albicans* dual-species biofilms were formed in selected media for 24 h and subsequently exposed for another 24 h to a combination of ciprofloxacin (CIP) and anidulafungin (AFG), at concentrations corresponding to 50× the MIC (minimum inhibitory concentration) for each agent. The MIC of CIP for the *S. epidermidis* strain was 0.256 mg/L, and the MIC of AFG for *C. albicans* was 0.03 mg/L. After exposure to the drugs, the metabolic activity of the cells in the biofilm was measured using the Alamar Blue^®^ assay. To avoid underestimation due to metabolically inactive persistent cells, a procedure of metabolic restoration was performed before the measurements were taken. For comparison, the metabolic activity of biofilm-forming cells cultivated for 24 h in each medium without treatment was also evaluated.

**Fig. 3:**
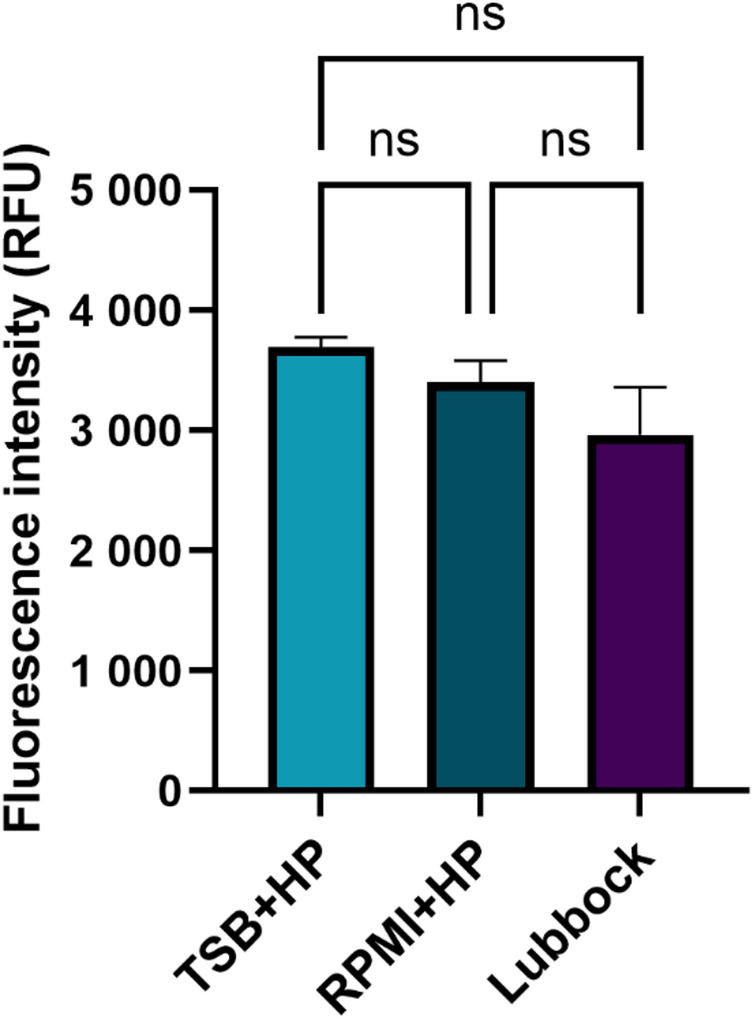
Metabolic activity of biofilm-forming agents in *S. epidermidis*-*C. albicans* dual-species consortia formed under different nutritional conditions. The total metabolic activity of *S. epidermidis* (ATCC 35983) and *C. albicans* (ATCC 90028) in 24-hour-old biofilms was determined using the metabolic indicator, Alamar Blue^®^. TSB + HP – Tryptic soy broth supplemented with 10% (v/v) human plasma (HP); RPMI + HP – RPMI 1640 supplemented with 10% (v/v) HP; Lubbock – Bolton broth supplemented with 50% (v/v) HP and 5% (v/v) freeze-thaw lysed sheep red blood cells. RFU – relative fluorescence units, ns – not significant. The values represent the mean ± SEM. Data were analysed using one-way ANOVA, and a *p*-value < 0.05 was accepted as statistically significant. TSB + HP vs. RPMI + HP: *p* = 0.9133; TSB + HP vs. Lubbock: *p* = 0.5166; RPMI + HP vs. Lubbock: *p* = 0.7174.

As shown in Fig. 3, no significant differences were revealed after analysis of the metabolic activity of the 24-hour-old biofilm-forming agents present within the biofilm consortia developed under different nutritional conditions without treatment. This finding is consistent with previous results (see Fig. 2), demonstrating no statistically significant differences in the multiplication ratios for both *S. epidermidis* and *C. albicans* present in biofilms formed in selected media, directly correlating with the number of biofilm-forming agents.

As shown in Fig. S2 (Supplementary information), subsequent exposure to the combination of CIP and AFG resulted in reduced metabolic activity in all *S. epidermidis*-*C. albicans* microbial biofilm communities formed in the selected media. The most pronounced decrease was observed in the biofilm communities formed in the Lubbock medium. However, the most statistically significant reduction of metabolic activity was registered for participants of biofilm formed in the TSB + HP medium.

**Fig. 4:**
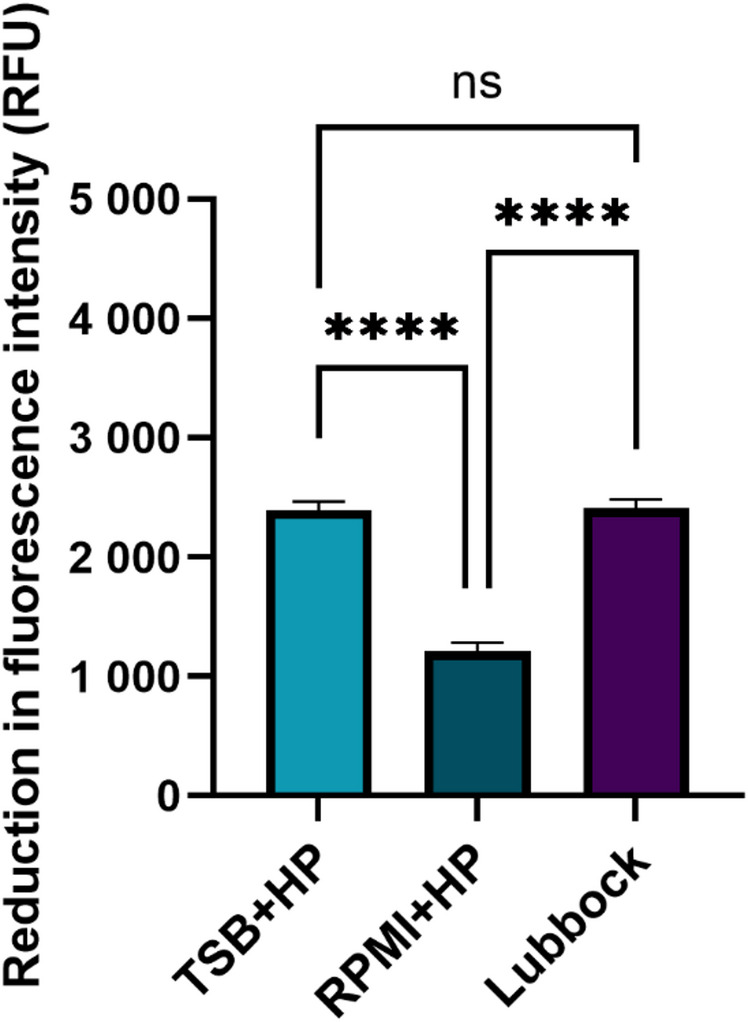
The difference in metabolic activity of biofilm-forming agents in *S. epidermidis*-*C. albicans* dual-species consortia formed under different nutritional conditions before and after exposure to selected antimicrobial agents. The reduction of metabolic activity of biofilm-forming agents was obtained from the comparison of the metabolic activity of *S. epidermidis* (ATCC 35983) and *C. albicans* (ATCC 90028) in dual-species biofilms formed in selected media before and after antimicrobial drug exposition. To calculate the reduction in fluorescence intensity, each value corresponding to the metabolic activity of participants of biofilms exposed to the combination of ciprofloxacin and anidulafungin was subtracted from the mean value corresponding to unexposed consortia. The values represent the mean ± SEM. Data were analysed using one-way ANOVA, and a *p*-value < 0.05 was accepted as statistically significant. TSB + HP vs. RPMI + HP: *p* < 0.0001; TSB + HP vs. Lubbock: *p* = 0.9846; RPMI + HP vs. Lubbock: *p* < 0.0001. TSB + HP – Tryptic soy broth supplemented with10% (v/v) human plasma (HP); RPMI + HP – RPMI 1640 supplemented with 10% (v/v) HP; Lubbock – Bolton broth supplemented with 50% (v/v) HP and 5% (v/v) freeze-thaw lysed sheep red blood cells; *RFU* relative fluorescence units; *ns* not significant.

A closer examination by comparative analysis of metabolic activity reduction after exposure to a combination of CIP and AFG among biofilm communities formed in selected media revealed that the lowest statistically significant decrease in metabolic activity was detected for communities formed in the RPMI + HP medium (see Fig. 4). Furthermore, no statistically significant difference in the reduction of metabolic activity was observed between the communities formed in either the TSB + HP or the Lubbock medium.

### Carbohydrates are the key matrix component in *S. epidermidis*-*C. albicans* dual-species biofilms despite formation under different nutritional conditions

Quantitative analyses of the biofilm matrix were performed to evaluate its potential contribution to antimicrobial tolerance, with a focus on its major components: carbohydrates, proteins, and extracellular DNA (eDNA). The 24-hour-old biofilms were homogenised, and the extracellular matrix fractions were used to quantify principal matrix biomolecules by commercially available kits.

**Fig. 5:**
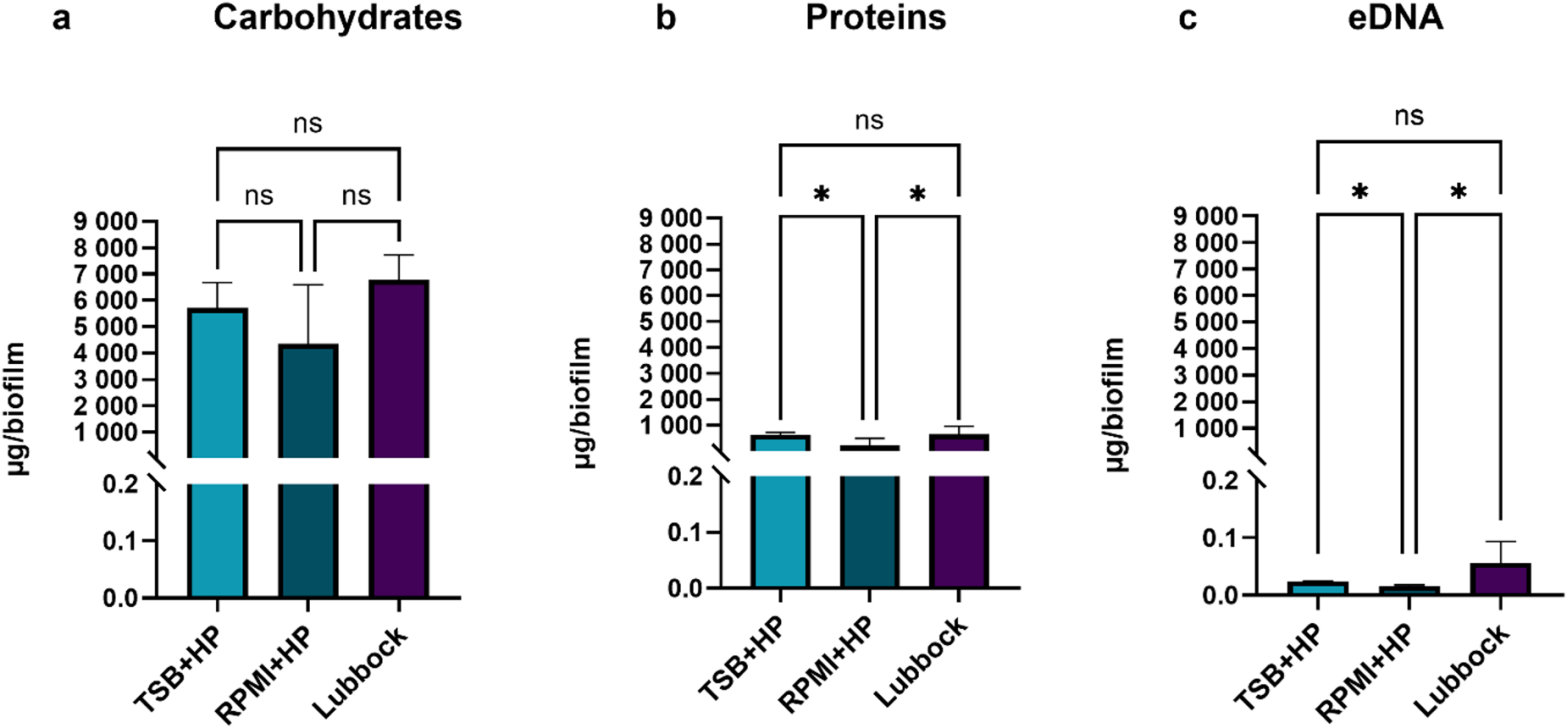
Comparison of key matrix macromolecule concentrations in *S. epidermidis*-*C. albicans* dual-species biofilms formed under different nutritional conditions. Dual-species *S. epidermidis* (ATCC 35983) and *C. albicans* (ATCC 90028) biofilm matrices were analysed after 24 h of biofilm formation in selected cultivation media: TSB + HP – Tryptic soy broth supplemented with 10% (v/v) human plasma (HP); RPMI + HP – RPMI 1640 supplemented with 10% (v/v) HP; Lubbock – Bolton broth supplemented with 50% (v/v) HP and 5% (v/v) freeze-thaw lysed sheep red blood cells. eDNA – extracellular DNA, ns – not significant. The values represent the mean ± SEM. Data were analysed using one-way ANOVA or Kruskal-Wallis test, and a *p*-value < 0.05 was accepted as statistically significant. **a** TSB + HP vs. RPMI + HP: *p* > 0.9999; TSB + HP vs. Lubbock: *p* = 0.3500; RPMI + HP vs. Lubbock: *p* = 0.13359. **b** TSB + HP vs. RPMI + HP: *p* = 0.0409; TSB + HP vs. Lubbock: *p* = 0.9974; RPMI + HP vs. Lubbock: *p* = 0.0115. **c** TSB + HP vs. RPMI + HP: *p* = 0.0105; TSB + HP vs. Lubbock: *p* > 0.9999; RPMI + HP vs. Lubbock: *p* = 0.0105.

As shown in Fig. S3 (Supplementary information), carbohydrates represented the most prevalent macromolecules in the biofilm matrix, with statistically significant predominance across all biofilms formed in the media used. When focusing on the quantitative differences of these macromolecules among individual biofilm biomasses formed under varying nutritional conditions, a statistically significant difference was observed only in proteins and eDNA content (Fig. 5). A statistically significantly higher proportion of proteins and eDNA was registered in biofilms developed in the TSB + HP and Lubbock media, representing communities with reduced tolerance to the combination of CIP and AFG (see Fig. 4).

### Epifluorescent and scanning electron microscopic analysis suggest differences in *S. epidermidis*-*C. albicans* dual-species biofilms formed under different nutritional conditions

For structural characterization of biofilm communities (spatial distribution, cell organisation and morphology, intercellular interactions, surface architecture, and biofilm matrix presence), epifluorescence microscopy using a combination of two fluorescent dyes, Calcofluor White and SYTO 9, and scanning electron microscopy were employed.

**Fig. 6:**
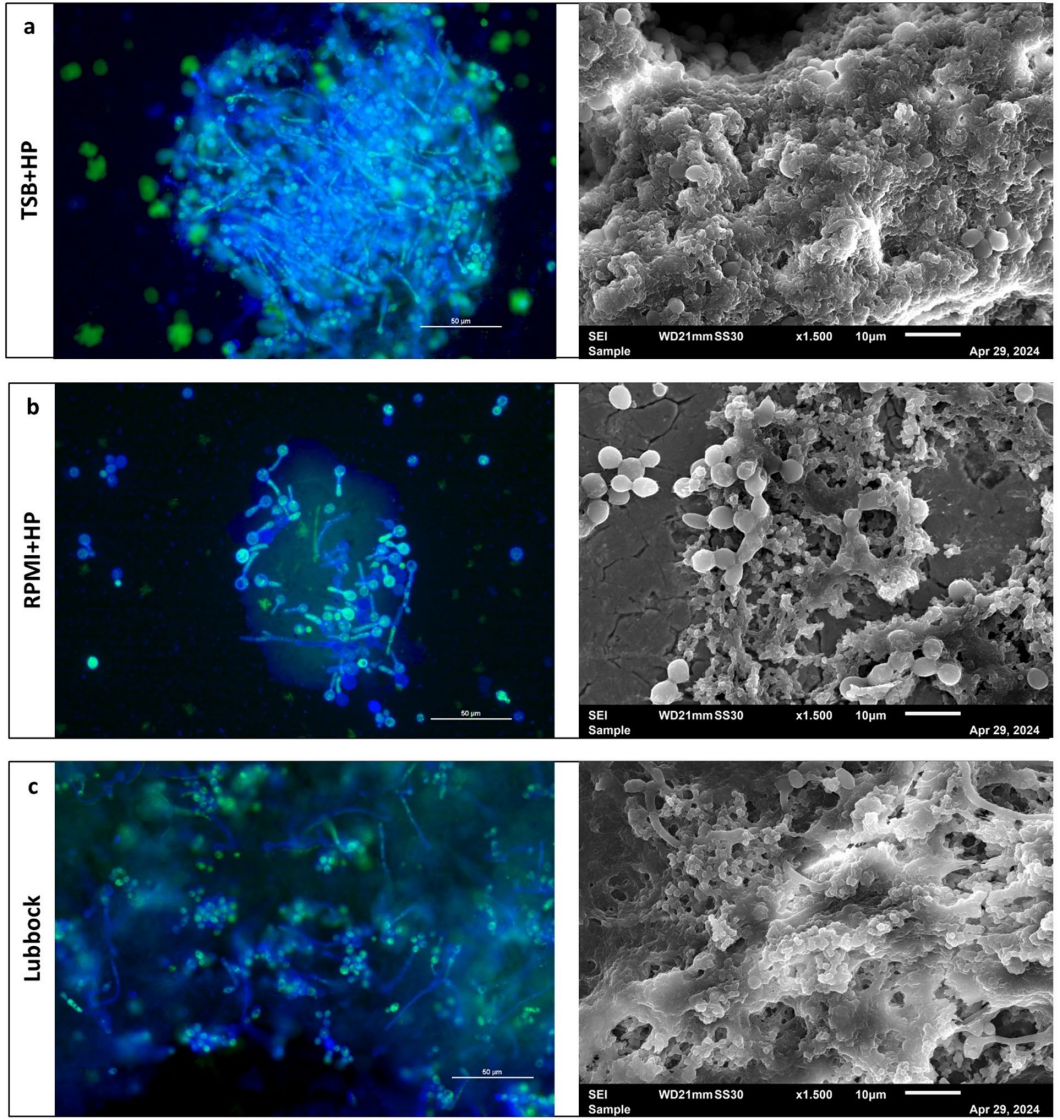
Epifluorescent and scanning electron microscopic images of *S. epidermidis*-*C. albicans* dual-species biofilms formed under different nutritional conditions. Dual-species *S. epidermidis* (ATCC 35983) and *C. albicans* (ATCC 90028) biofilm consortia were formed for 24 h in the selected cultivation media: (a) TSB + HP – Tryptic soy broth supplemented with 10% (v/v) human plasma (HP); (b) RPMI + HP – RPMI 1640 supplemented with 10% (v/v) HP; (c) Lubbock – Bolton broth supplemented with 50% (v/v) HP and 5% (v/v) freeze-thaw lysed sheep red blood cells. The combination of two fluorescent dyes, Calcofluor White and SYTO 9, was used for epifluorescent microscopy of microbial cells (blue – yeast cells, green – bacterial cells) and biofilm matrix (blue, green stained unbounded, dispersed mass) visualisation. The scale bar corresponds to 50 μm. In scanning electron microscopic images, larger oval cells and hyphae represent yeast, and smaller round cells indicate bacteria. The biofilm matrix appears as an amorphous substance covering cells. The scale bar corresponds to 10 μm.

As shown in Fig. 6, the transition of the yeast *C. albicans* to a pseudohyphal or hyphal form was observed in all biofilm communities formed in the selected media. A visually more complex biofilm architecture appeared to develop in the consortia formed in the TSB + HP and Lubbock medium. Likewise, both types of microscopical inspection qualitatively suggest that the biofilm matrix more extensively covers the biofilm structures in communities formed in the TSB + HP and Lubbock medium. To support these observations, image analysis of fluorescence microscopy images was performed (Fig S4, Supplementary Information). Biofilms formed in TSB + HP and Lubbock medium exhibited a significantly higher integrated fluorescence density compared to those formed in RPMI + HP. These observations are in agreement with the increased wet and dry weight measurements of *S. epidermidis*-*C. albicans* dual-species biofilms, as well as the corresponding biomass surface density (Table S3, Supplementary Information). However, it should be noted that total biomass estimation based on crystal violet staining and the calculated multiplication ratios do not fully correlate with the weight-based measurements. This discrepancy likely reflects methodological differences and limitations.

## Discussion

*S. epidermidis* and *C. albicans* are integral members of the human commensal microbiome and act as prominent opportunistic pathogens, frequently implicated in endogenous infections, such as wound infections and catheter-associated bloodstream infections. Their coexistence in the host microbiome increases the risk of polymicrobial infections, including biofilm-associated infections^1,2^.

In this study, we aimed to assess, analogously to our previous findings for MRSA-*C. albicans* dual-species biofilm consortia^24^, whether the robustness and heterogeneity of microbial communities, enhanced by a greater abundance of HP proteins, would correlate with the ability of the biofilm-forming species, *S. epidermidis* and *C. albicans*, to withstand the action of selected antimicrobial agents.

Several well-established mechanisms may underpin a potential linear correlation between biofilm biomass robustness, structural heterogeneity, and the reduced antimicrobial susceptibility^34^. Increased biofilm biomass can be associated not only with a higher density of microbial cells but also with an expanded extracellular matrix. This matrix serves as both a physical and chemical barrier, impeding antimicrobial diffusion and potentially inactivating or sequestering antimicrobial agents^10^. Furthermore, greater biomass contributes to a more intricate three-dimensional architecture, establishing microenvironmental gradients in the deeper layers. These gradients promote physiological heterogeneity among biofilm microbial participants, which allow them to adapt to nutrient- and oxygen-limited conditions by reducing metabolic activity and entering a dormant state^10,34^.

Conversely, the observed correlation between biofilm biomass robustness and antimicrobial tolerance cannot be generalized across all biofilm communities. The response of biofilm-forming microbes to antimicrobial exposure is governed by a complex interplay of multiple factors, including environmental conditions, nutrient availability, and host-derived factors^35^. Accordingly, we focused on the role of host soluble factors in HP and FT-RBC, on the properties of in vitro formed *S. epidermidis*-*C. albicans* dual-species biofilm consortia, with particular emphasis on their capacity to withstand environmental stress induced by a selected combination of antimicrobial agents.

Firstly, we demonstrated that the *S. epidermidis* and *C. albicans* cooperation in dual-species biofilms enhances the total biofilm biomass across all included cultivation media compared to the mono-species biofilms. Notably, *S. epidermidis* alone was not able to establish a biofilm community in RPMI + HP and the Lubbock media, and was recognised only as a weak biofilm producer in TSB + HP. This contrasts with our previous findings^36^, where the same strain was classified as a strong biofilm producer under similar conditions. The key difference may lie in the use of heat-inactivated HP in the previous study, suggesting that heat-labile components in HP may contribute to the reduced biofilm-forming capacity of *S. epidermidis* observed in our present study.

The greatest amount of *S. epidermidis*-*C. albicans* dual-species biofilm biomass was formed in the Lubbock medium. Total biofilm biomass comprises both the microbial biofilm participants as well as the extracellular biofilm matrix. The glue-like matrix is not solely composed of molecules secreted by biofilm-forming microorganisms. It may also entrap environmental macromolecules and host cell debris^37,38^. Therefore, the higher proportion of host soluble factors in HP and FT-RBC present in the Lubbock medium may contribute to the increased biofilm biomass.

Following this, we questioned whether the formation of greater amounts of more robust and complex *S. epidermidis*-*C. albicans* dual-species consortia would lead to higher levels of antimicrobial tolerance. The strongest ability to withstand environmental stress induced by the action of the selected antimicrobial drug combination was seen in the *S. epidermidis*-*C. albicans* dual-species biofilms formed in the RPMI + HP medium. In contrast, communities formed in the TSB + HP and Lubbock media had reduced metabolic activity, reflecting lower resilience. Microscopy revealed that biofilms formed in both the TSB + HP and Lubbock medium were more heterogeneous and structurally complex. Thus, our findings do not support the direct correlation between dual-species *S. epidermidis*-*C. albicans* biofilm robustness or complexity and antimicrobial tolerance.

This raises the question whether the different levels of host soluble factors in HP or FT-RBC could explain the variability in *S. epidermidis*-*C. albicans* dual-species biofilms antimicrobial tolerance. Dual-species *S. epidermidis*-*C. albicans* biofilms formed in the TSB + HP and Lubbock media showed reduced antimicrobial tolerance compared to consortia formed in RPMI + HP medium. Since the TSB + HP and RPMI + HP media contained an equal amount of HP, whereas the Lubbock medium contained a five times higher amount of HP and additionally FT-RBC, it suggests that neither HP, nor FT-RBC plays a pivotal role in the antimicrobial tolerance mechanisms of *S. epidermidis*-*C. albicans* dual-species biofilms against selected antimicrobial drugs. Likewise, no effect of HP or FT-RBC on the proliferative capacity of *S. epidermidis* and *C. albicans* in dual-species consortia was observed.

In contrast, our previous study of MRSA-*C. albicans* dual-species communities^24^ revealed a distinct role of HP. The Lubbock medium led to the formation of biofilm consortia with the highest level of antimicrobial tolerance. However, this finding should not be unexpected. Unlike *S. epidermidis*, MRSA produces virulence factors, like coagulase, which converts plasma proteins into clots. It contributes to the formation of a dense, semi-solid shield protecting microbial communities from hostile conditions, including the action of antimicrobials^39^.

The pivotal role in the antimicrobial tolerance of microorganisms in biofilms is represented by the biofilm matrix, acting as a physical barrier that restricts antimicrobial penetration and contributes to their inactivation^40,41^. In our study, carbohydrates were the predominant components in the *S. epidermidis*-*C. albicans* dual-species biofilm matrices across all selected media. This dominance has also been demonstrated for mono-species *S. epidermidis* and *C. albicans* biofilms in other published studies^42–46^, as well as for MRSA-*C. albicans* dual-species communities under identical conditions in our previous study^24^. Statistically significant higher proteins and eDNA concentrations were found in biofilm matrices formed in media with a higher availability of nitrogen sources (TSB + HP and Lubbock), compared to those formed in the RPMI + HP medium with reduced nitrogen availability. However, since communities formed in RPMI + HP medium showed a greater ability to withstand environmental stress, induced by the action of a combination of selected antimicrobial drugs, matrix proteins and eDNA are unlikely to play a crucial role in antimicrobial tolerance.

Thus, what accounts for the higher antimicrobial tolerance of *S. epidermidis* and *C. albicans* in dual-species biofilms formed in medium with reduced nitrogen availability? We propose that nutrient limitation induces environmental stress. Activating stress response mechanisms enhances the ability to better withstand environmental challenges, including antimicrobial exposure. Furthermore, we hypothesise that in nitrogen-rich media, elevated nitrogen consumption may lead to the accumulation of ammonia byproducts, creating neutral or slightly alkaline niches within the biofilms, while nitrogen limitation leads to a predominance of acidic biofilm microenvironments^47–49^. This hypothesis is consistent with our experimental observations (Table S3, Supplementary information), where the pH of *S. epidermidis*-*C. albicans* dual-species biofilms formed in different cultivation media was measured. As microbial metabolism, membrane permeability, and the efficacy of many antimicrobials are pH-dependent, it could affect the response to treatment^50–52^. The effectiveness of CIP, used in this study, is reduced under acidic pH levels^53,54^. A similar trend may apply to AFG; however, published studies about AFG explicitly addressing the role of pH modulating its antimicrobial efficacy are currently lacking^55^.

In conclusion, we demonstrate that an increased level of host soluble factors in HP and FT-RBC leads to the formation of a higher amount of complex *S. epidermidis*-*C. albicans* dual-species biofilm biomass in vitro. Nevertheless, the robustness of these biofilm consortia did not correlate with their degree of antimicrobial tolerance. In contrast, a higher level of *S. epidermidis*-*C. albicans* dual-species biofilm antimicrobial tolerance was detected under nitrogen-limited conditions. In the context of in vitro dual-species biofilm formation studies aimed at developing effective treatment strategies for biofilm-associated infections, our findings highlight the importance of considering host-specific nutritional niche conditions at the site of infection and species-specific characteristics, as even phylogenetically related microorganisms may display markedly distinct physiological and adaptive behaviours.

## Methods

### Microbial strains

The reference microbial strains included in previous published studies^36,56^: bacterium *S. epidermidis* (ATCC 35983; CCM 7844), and yeast *C. albicans* (ATCC 90028; CCM 8261), were purchased from the Czech Collection of Microorganisms (CCM, Czech Republic). Before each experiment, cryopreserved stocks of bacterial/yeast suspensions were inoculated on Mueller-Hinton agar/Sabouraud dextrose agar (Himedia, India) and incubated at 37 °C in a dark, humid atmosphere for 16–24 h.

### Cultivation media

Three nutritionally different media were used in the study. Specifically: Tryptic soy broth (TSB; Himedia, India), supplemented with 10% (v/v) human plasma (HP, Biowest, France), pH 7.3 ± 0.2; a chemically defined RPMI 1640 medium with L-glutamine, sodium bicarbonate, and D-glucose (2 g/L) (RPMI; Merck, USA), supplemented with 10% (v/v) HP, pH 7.4 ± 0.2; and Bolton broth (Merck, USA) supplemented with 50% (v/v) HP, and 5% (v/v) freeze-thaw lysed sheep red blood cells (FT-RBC; LabMediaServis, Czech Republic), pH 7.4 ± 0.2, which is a slight modified version of the Lubbock medium^25^ (with sheep erythrocytes instead of horse).

### Formation of *S. epidermidis/C. albicans* mono-species and *S. epidermidis*-*C. albicans* dual-species biofilms in vitro

In individual cultivation media, homogenous suspensions of bacteria/yeasts with an optical density (O.D., 565±15 nm, DEN-1, BioSan, Latvia) of 0.5 McFarland units were prepared. The microdilution method and spread plate technique were employed to evaluate CFU/mL in the initial inocula. The number of bacterial cells in the homogenous suspensions (O.D. = 0.5 McFarland units) corresponded to approximately 1.15⋅10^7^ CFU/mL. The number of yeast cells in the homogenous suspensions (O.D. = 0.5 McFarland units) corresponded to approximately 1.07⋅10^6^ CFU/mL.

Biofilms were formed in a 96-well microtiter plate previously coated with HP: All wells were filled with 200 µL of HP and incubated at 37 °C, in a humid atmosphere for 24 h. After incubation, the HP was removed, and the wells were left to air dry.

To form both *S. epidermidis* and *C. albicans* mono-species biofilms, each well was filled with 200 µL of homogeneous microbial suspensions in appropriate cultivation media. For the formation of *S. epidermidis*-*C. albicans* dual-species biofilms, each well was filled with 100 µL of homogenous bacterial suspensions and 100 µL of homogeneous yeast suspensions in appropriate cultivation media. The plates were then incubated at 37 °C for 24 h in a humid atmosphere on a rocking Table (25 rpm, Mini Rocker Shaker MR-1, BioSan, Latvia).

### Quantification of *S. epidermidis*/*C. albicans* mono-species and *S. epidermidis*-*C. albicans* dual-species total biofilm biomass and characterisation of phenotypic producers by crystal violet staining

For the quantification of the total biofilm biomasses and biofilm producer phenotype determination, the method put forth by Christensen et al.^32^ was used. The 24-hour-old biofilms were rinsed three times with a sterile 0.9% saline solution, air-dried, and fixed with ice-cold methanol for 15 min at 4 °C. The fixed biofilms were incubated with 200 µL of a 0.05% CV solution per well for 30 min at room temperature on a rocking table. Afterward, all excess stain was removed, and the biofilms were rinsed three times with deionised water. The retained CV was eluted using an ethanol:acetone mixture (80:20, v/v) at room temperature for 30 min on a rocking table. Finally, the O.D. of the eluted CV was measured at 570 nm using a Synergy HTX Multimode microplate reader (BioTek, USA).

Based on the O.D. of the eluted CV (O.D._CV_), the microorganisms forming mono- and dual-species biofilms were classified into biofilm producer phenotypes according to the criteria described by Stepanović et al.^33^. Strains were classified as non-biofilm producers (-) if O.D._CV_ ≤ O.D._C_ (O.D. of negative control). Strains with O.D._C_ < O.D._CV_ ≤ 2×O.D._C_ were categorised as weak biofilm producers (+). Those with 2×O.D._C_ < O.D._CV_ ≤ 4×O.D._C_ were considered moderate biofilm producers (++). Ultimately, strains with 4×O.D._C_ < O.D._CV_ were classified as strong biofilm producers (+++).

### Evaluation of the metabolic activity of biofilm-forming agents in *S. epidermidis*-*C. albicans* dual-species consortia before and after exposure to antimicrobial drugs

To determine the ability of microbial participants in biofilms to withstand adverse hostile conditions, commercially available antimicrobials, fluoroquinolone antibiotic ciprofloxacin (CIP) and echinocandin antimycotic anidulafungin (AFG) (both Merck, USA) were employed. The first step was to determine the MIC for the planktonic forms of *S. epidermidis* (MIC_CIP_) and *C. albicans* (MIC_AFG_) using the microdilution broth method according to EUCAST guidelines^57,58^. Dimethyl sulfoxide was used as the cosolvent for the antimicrobials, with the final concentration not exceeding 1% (v/v).

The next step involved exposing the biofilms to 50×MIC of CIP and AFG as follows: 24-hour-old biofilms were rinsed three times with sterile 0.9% saline solution. Then, 100 µL of CIP solution in Cation-adjusted Mueller-Hinton broth (CAMHB, M-H 2 Broth, Merck, United States) at a final concentration of 12.8 mg/L, and 100 µL of AFG solution in the RPMI 1640 medium supplemented with L-glutamine, sodium bicarbonate, and D-glucose (2 g/L) at a final concentration of 1.5 mg/L, were added into each well, and incubated at 37 °C in a humid atmosphere for 24 h.

Following this, to restore the metabolic activity of persisting cells, the media containing antimicrobials were replaced with a fresh TSB medium, the biofilms were subjected to two sonication steps (each lasting 5 min) and incubated for 75 min at 37 °C in a humid atmosphere. Afterward, 20 µL of a solution of the metabolic indicator Alamar Blue^®^ Cell Viability reagent (Thermo Fisher Scientific) was added to each well. After 30 min of incubation at 37 °C in a humid atmosphere and a gentle shaking mode, fluorescence intensity was measured at λ_Ex_ 530 nm and λ_Em_ 590 nm using a Synergy HTX Multimode microplate reader. For comparison, the metabolic activity of biofilm-forming participants without any drug exposure was processed in the same manner. Briefly, 24-hour-old biofilms were rinsed, a fresh TSB medium was added, and the biofilms were subjected to sonication and incubation steps. The Alamar Blue^®^ solution was then added, and the fluorescence intensity was measured (all under the same conditions as for the biofilms exposed to antimicrobials).

### Extraction and quantification of biofilm-forming agents and key matrix biomolecules in the *S. epidermidis*-*C. albicans* dual-species consortia

For the extraction and quantification of microbial multiplication ratios and key matrix components, *S. epidermidis*-*C. albicans* dual-species biofilms were formed in 24-well microtiter plates previously coated with HP. Each well was filled with 0.9 mL of a homogeneous bacterial suspension and 0.9 mL of a yeast homogeneous suspension (both O.D. = 0.5 McFarland units). The biofilm-forming participants were then cultivated under the same conditions as described above. After 24 h of incubation, the biofilms were rinsed three times with sterile 0.9% saline solution and air-dried. Then, 0.5 mL of a sterile 0.9% saline solution was added to each well, and the biofilms were carefully scraped, transferred into a Falcon tube, and homogenised for 1 min using a Handheld Homogeniser (MT-30 K, Miulab, China) at 18 000 rpm.

Carbohydrate and protein components of the biofilm matrices from mechanically homogenised biofilms were subsequently disintegrated using α-amylase from *Aspergillus oryzae* and proteinase K from *Tritirachium album* (both Merck, USA). First, α-amylase was added at a final concentration of 20 mg/mL in 20 mM Tris-HCl buffer with 100 mM NaCl (pH 7.4) and incubated for 45 min at 37 °C. Then, proteinase K was added at a final concentration of 200 µg/mL in 20 mM Tris-HCl buffer with 3 mM CaCl_2_ (pH 7.4) and incubated for another 45 min at 37 °C. This was followed by two sonication steps (each lasting 5 min) and centrifugation (Hettich, Germany) at 10 000 × g for 15 min at 24 °C. Finally, the supernatants were transferred to test tubes as extracellular matrix fractions, which were subsequently filter-sterilised (0.22 μm pore size).

Pelleted microorganisms were resuspended in a sterile 0.9% saline solution, serially diluted, and seeded on Mueller-Hinton agar and Sabouraud dextrose agar. After 24 h of cultivation at 37 °C, the CFU/mL were counted.

For the evaluation of multiplication ratios, the initial CFU/mL were processed in the same manner. Briefly, initial microbial suspensions were serially diluted and seeded on Petri dishes containing growth media. After 24 h of cultivation at 37 °C, the CFU/mL were counted. Multiplication ratios were then calculated as the ratio between the CFU/mL present in the biofilm consortia after 24 h of cultivation and the CFU/mL present at the beginning of cultivation.

The filter-sterilized extracellular matrix fractions were used for the quantification of key matrix components. The following commercially available kits were employed: Total Carbohydrate Assay Kit (Merck, USA) for carbohydrates; Pierce™ BCA Protein Assay Kit (Thermo Fisher, USA) for proteins; and Quant-iT™ PicoGreen™ dsDNA Reagent and Kit (Thermo Fisher, USA) for eDNA. All analyses were performed according to the manufacturer’s instructions.

### Epifluorescent microscopy of *S. epidermidis*-*C. albicans* dual-species biofilms

The 24-hour-old biofilms were rinsed three times with a sterile 0.9% saline solution. The fluorescent stain SYTO 9 (visualisation of the DNA of living cells and eDNA in the biofilm matrix; Merck, USA) was added to each well at a final concentration of 4.90 µM to completely cover the biofilms, followed by a 30-minute incubation in the dark with gentle shaking at room temperature. Afterward, the biofilms were scraped off, transferred to a microscopic slide, and two drops of Calcofluor white (visualisation of yeasts and polysaccharides in the biofilm matrix; Merck, USA) were added. Finally, the sample was covered with a cover slip. The stained biofilms were imaged using an Olympus Provis fluorescent microscope (Olympus, Japan) equipped with a photographic device (DS-Fi3, Nikon, Japan). The images were collected and adjusted using NIS-Elements software, version 5.00 (Laboratory Imaging, Czech Republic).

### Scanning electron microscopy of *S. epidermidis*-*C. albicans* dual-species biofilms

For the visualisation of biofilms using scanning electron microscopy (SEM), *S. epidermidis*-*C. albicans* dual-species biofilms were formed in 48-well microtiter plates on stainless steel discs (DIN9021, stainless steel A2, size M2, diameter 5.9 mm) previously coated with HP, under the same conditions as described above. Each well was filled with 250 µL of homogeneous bacterial suspension and 250 µL of yeast homogeneous suspension (both O.D. = 0.5 McFarland units). The 24-hour-old biofilms were rinsed three times with sterile 0.9% saline solution and fixed overnight in 2.5% glutaraldehyde at 4 °C. The fixed biofilms on the metal discs were then dehydrated using a series of increasing ethanol concentrations (50%, 70%, 80%, and 99.9%), with 5-minute incubations at each concentration. The metal discs with the dehydrated biofilms were fixed onto aluminium pins using Leit-C gluing strips (Göcke, Plano GmbH, Wetzlar, Germany) and coated with gold for 40 s using an Agar Sputter Coater (Agar Scientific Ltd., Rotherham, UK). Finally, the biofilms were imaged using SEM (JSM-6010LV, JEOL GmbH, Freising, Germany).

### Statistical analysis

Data were collected from three independent experiments, with at least six replicates in each. The O.D. and relative fluorescent unit values were corrected by subtracting the equivalent values from negative controls (individual cultivation media). Statistical analysis was performed using GraphPad Prism software version 10.6.1 (GraphPad Software, Inc., USA). Data were first tested for normality and lognormality. Normally distributed datasets were analysed using one-way ANOVA followed by Tukey’s multiple comparisons test. Non-normally distributed datasets were analysed using the Kruskal-Wallis test followed by Dunn’s multiple comparisons test. For comparisons involving only two groups, unpaired *t*-tests were performed. The results were considered statistically significant when the *p*-value was < 0.05. Data are presented as the mean ± SEM.

## Supplementary Information

Below is the link to the electronic supplementary material.

Supplementary Material 1

## Data Availability

The data supporting the findings of this study are available within the article and in the Supplementary information. Other data related to this study are available from the corresponding author upon reasonable request.
