# Supplementary material for: Host soluble plasma factors increase dual-species Staphylococcus epidermidis and Candida albicans biofilm biomass without enhancing stress tolerance
Source: Sci Rep. 2026 Apr 22;16:18758. doi: 10.1038/s41598-026-49557-1 (PMC13273201; doi:10.1038/s41598-026-49557-1)
Supplement: Supplementary file 1 — Supplementary Material 1 [file 41598_2026_49557_MOESM1_ESM.pdf]

Host soluble plasma factors increase dual-species *Staphylococcus epidermidis* and *Candida albicans* biofilm biomass without enhancing stress tolerance

Pavlína Vávrová[a], Ondřej Janďourek[a], Débora Cristina Coraça-Huber[b], Christopher Spiegel[b], Petr Nachtigal[a], Martin Krátký[c], Klára Konečná[a#]

[a]Charles University, Faculty of Pharmacy in Hradec Králové, Department of Biological and Medical Sciences, Akademika Heyrovského 1203/8, 500 03, Hradec Králové, Czech Republic

[b]University Hospital for Orthopaedics and Traumatology, Medical University of Innsbruck, Research Laboratory for Biofilms and Implant Associated Infections (BIOFILM LAB), Müllerstraße 44, 6020 Innsbruck, Austria

[c]Charles University, Faculty of Pharmacy in Hradec Králové, Department of Organic and Bioorganic Chemistry, Akademika Heyrovského 1203/8, 500 03 Hradec Králové, Czech Republic

[#]Address correspondence to Klára Konečná, konecna@faf.cuni.cz

Charles University, Faculty of Pharmacy in Hradec Králové, Department of Biological and Medical Sciences, Akademika Heyrovského 1203/8, 500 03, Hradec Králové, Czech Republic; Tel: + (420) 495 067 366, ORCID:0000-0001-5670-7767

Supplementary information

Table S1: Key nutrients availability in cultivation media for *in vitro* biofilm formation

	TSB+HP	RPMI+HP	Lubbock
Basic medium	Tryptic soy broth	RPMI 1640	Bolton broth
HP supplementation	10%	10%	50%
FT-RBC supplementation	-	-	5%
Proteins/peptides/amino acids (g/L)	25.00-28.00	6.00-9.00	62.50-77.50
Glucose (g/L)	2.60-2.64	2.10-2.14	0.59-0.81
Final pH	7.3±0.2	7.4±0.2	7.4±0.2

Footnote: HP – human plasma; FT-RBC – freeze-thaw lysed sheep red blood cells; TSB+HP – Tryptic soy broth supplemented with 10% (v/v) HP; RPMI+HP – RPMI 1640 medium supplemented with 10% (v/v) HP, Lubbock – Bolton broth medium supplemented with 50% (v/v) HP and 5% (v/v) FT-RBC.

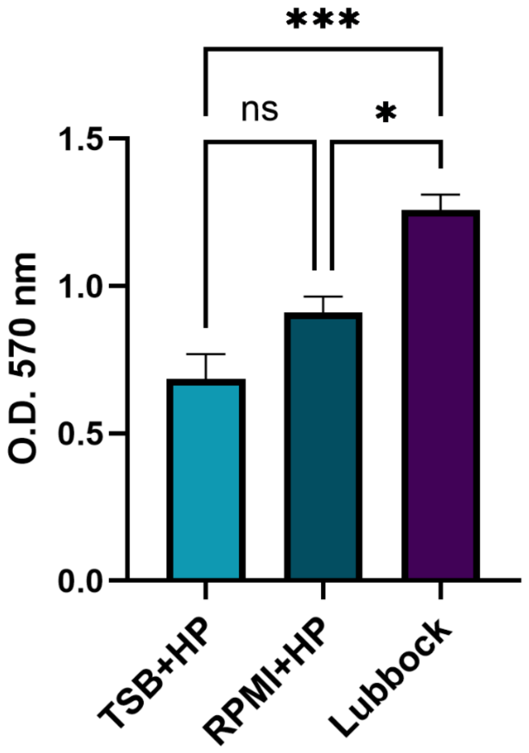

Fig. S1: Comparison of total *S. epidermidis-C. albicans* dual-species biofilm biomass formed under different nutritional conditions

Dual-species biofilms of *S. epidermidis* (ATCC 35983) and *C. albicans* (ATCC 90028) were cultivated *in vitro* in different cultivation media for 24 hours. TSB+HP – Tryptic soy broth + 10% (v/v) human plasma (HP), RPMI+HP – RPMI 1640 + 10% (v/v) HP, Lubbock – Bolton broth medium supplemented with 50% (v/v) HP and 5% (v/v) FT-RBC. The crystal violet staining method was employed for the quantification of total biofilm biomass. The values represent the mean ± SEM. Data were analysed using the Kruskal-Wallis test, and a *p*-value < 0.05 was accepted as statistically significant. TSB+HP vs RPMI+HP: $p = 0.6063$; TSB+HP vs Lubbock: $p = 0.0003$; RPMI+HP vs Lubbock $p = 0.0361$. O.D. – optical density; ns – not significant.

Table S2: Relative abundance of *S. epidermidis* and *C. albicans* microbial cells in *S. epidermidis*-*C. albicans* dual-species biofilms formed in different cultivation media

Cultivation medium	*S. epidermidis* (%)	*C. albicans* (%)
TSB+HP	44.95-55.72	44.28-55.05
RPMI+HP	50.69-56.43	43.57-49.31
Lubbock	33.79-57.23	42.77-66.42

Footnote: Relative abundance of microbial cells within the biofilm consortia was determined as the log-transformed proportion (%) of each species, *S. epidermidis* (ATCC 35983) and *C. albicans* (ATCC 90028), relative to the total number of microbial cells present in the dual-species biofilms after 24 hours of cultivation in different cultivation media: TSB+HP – Tryptic soy broth supplemented with 10% (v/v) human plasma (HP); RPMI+HP – RPMI 1640 supplemented with 10% (v/v) HP; Lubbock – Bolton broth supplemented with 50% (v/v) HP and 5% (v/v) freeze-thaw lysed sheep red blood cells. Data are presented as calculated ranges.

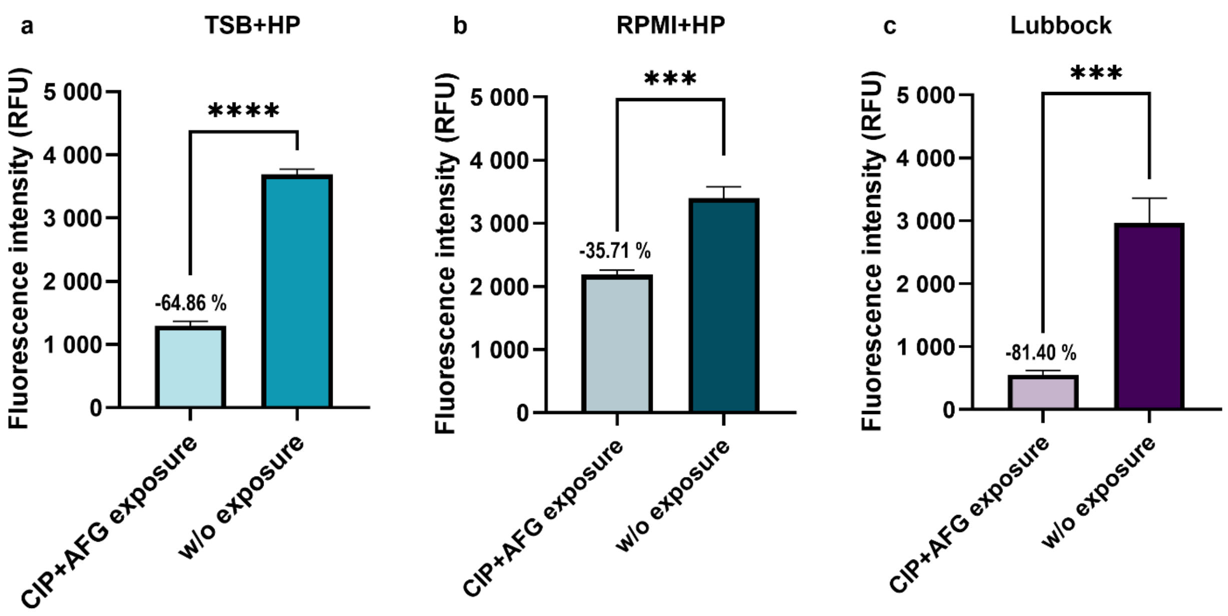

Fig. S2: Metabolic activity of biofilm-forming agents in *S. epidermidis*-*C. albicans* dual-species consortia before and after antimicrobial drugs exposition

The indicator, Alamar Blue®, was employed for the determination of metabolic activity of *S. epidermidis* (ATCC 35983) and *C. albicans* (ATCC 90028) in 24-hour-old dual-species biofilms before antimicrobial drugs exposure (w/o exposure) and after the next 24-hour exposure to ciprofloxacin and anidulafungin (CIP+AFG exposure). The values represent the mean ± SEM. The percentages above the columns express the difference in metabolic activity of biofilm-forming microbial participants after antimicrobial drugs exposure, related to the metabolic activity of unexposed biofilm-forming microbial participants before drug exposure (100%). Data were analysed using a *t*-test, and a p-value < 0.05 was accepted as statistically significant. a) TSB+HP – Tryptic soy broth supplemented with 10 % (v/v) human plasma (HP): $p < 0.0001$; b) RPMI+HP – RPMI 1640 supplemented with 10% (v/v) HP: $p = 0.0003$; c) Lubbock – Bolton broth supplemented with 50% (v/v) HP and 5 % (v/v) freeze-thaw lysed sheep red blood cells: $p = 0.0001$. RFU – relative fluorescence units.

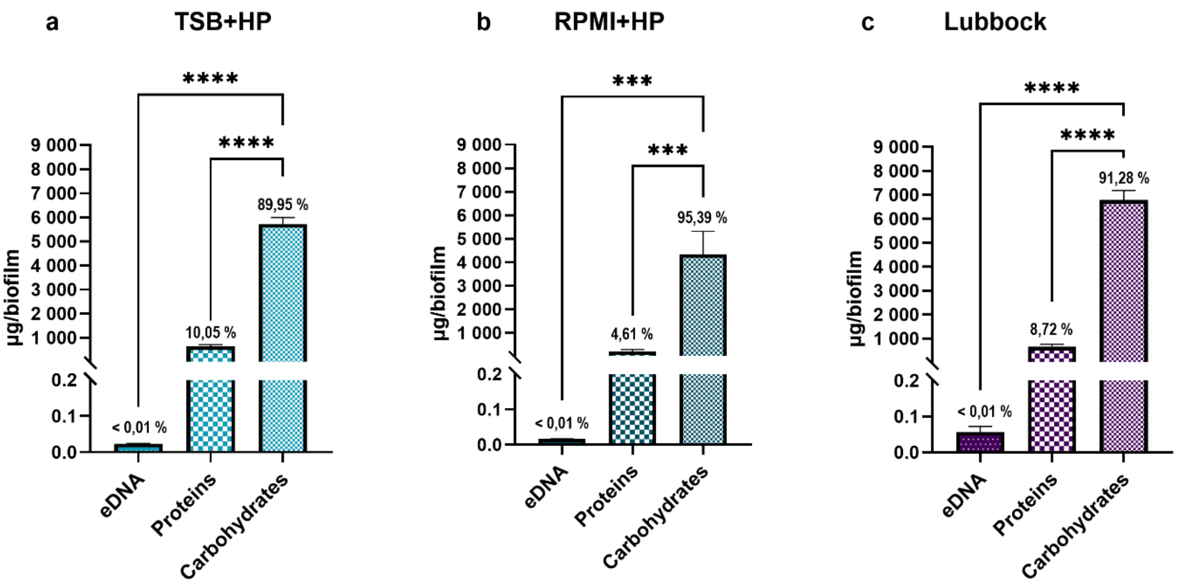

Fig. S3: Key biofilm matrix macromolecules in *S. epidermidis*-*C. albicans* dual-species biofilms formed under different nutritional conditions

S. epidermidis (ATCC 35983) and *C. albicans* (ATCC 90028) dual-species biofilms were formed for 24 hours in selected cultivation media. The values represent the mean ± SEM. The percentages above the columns express the weight amount related to total macromolecule mass (sum of proteins, carbohydrates, and extracellular DNA (eDNA) masses). To compare the dominance of carbohydrates with proteins and eDNA, data were analysed using *t*-test, and a p-value < 0.05 was accepted as statistically significant. a) TSB+HP – Tryptic soy broth supplemented with 10% (v/v) human plasma (HP): eDNA vs Carbohydrates: $p < 0.0001$; Proteins vs Carbohydrates: $p < 0.0001$; b) RPMI+HP – RPMI 1640 supplemented with 10% (v/v) HP: eDNA vs Carbohydrates: $p = 0.0001$; Proteins vs Carbohydrates: $p = 0.0002$; c) Lubbock – Bolton broth supplemented with 50% (v/v) HP and 5% (v/v) freeze-thaw lysed sheep red blood cells: eDNA vs Carbohydrates: $p < 0.0001$; Proteins vs Carbohydrates: $p < 0.0001$.

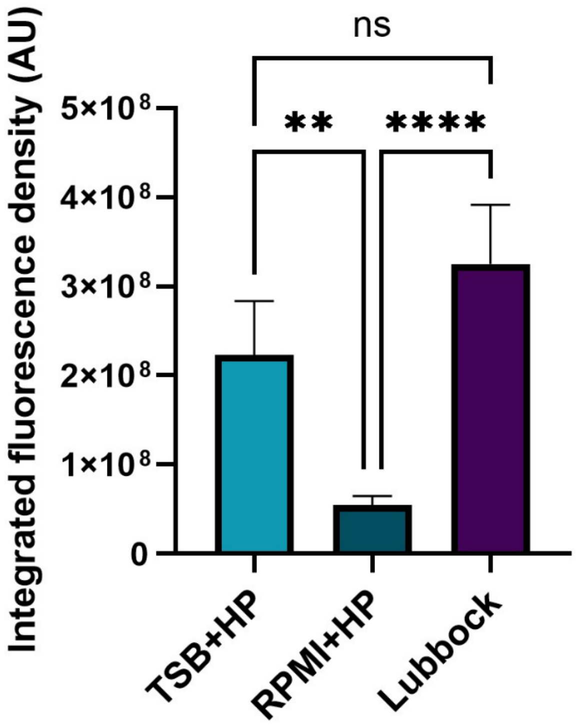

Fig. S4: Comparison of the integrated fluorescence density of *S. epidermidis*-*C. albicans* dual-species biofilms formed under different nutritional conditions

Dual-species biofilms of *S. epidermidis* (ATCC 35983) and *C. albicans* (ATCC 90028) were cultivated *in vitro* in different cultivation media for 24 hours. TSB+HP – Tryptic soy broth + 10% (v/v) human plasma (HP), RPMI+HP – RPMI 1640 + 10% (v/v) HP, Lubbock – Bolton broth medium supplemented with 50% (v/v) HP and 5% (v/v) FT-RBC. Biofilms were stained using a combination of Calcofluor White and SYTO 9 and visualized by epifluorescence microscopy (Olympus Provis fluorescent microscope, Olympus, Japan). For quantitative image analysis, five independent samples for each dual-species biofilm in the appropriate cultivation medium were prepared, and three random images were captured from each sample using a photographic device (DS-Fi3, Nikon Japan). The images were then analysed using NIS-Elements software, version 5.00 (Laboratory Imaging, Czech Republic). The integrated fluorescence density served as the quantitative parameter for subsequent comparative analysis. The resulting data were statistically analysed using GraphPad Prism software version

10.6.1. Data are presented as mean ± SEM. Data were analysed using the one-way ANOVA, and a *p*-value < 0.05 was accepted as statistically significant. TSB+HP vs RPMI+HP: *p* = 0.0003; TSB+HP vs Lubbock: *p* = 0.8131; RPMI+HP vs Lubbock *p* < 0.0001. ns – not significant, AU – arbitrary units.

Table S3: Physiochemical properties of *S. epidermidis-C. albicans* dual-species biofilm biomass formed in different cultivation media

	TSB+HP	RPMI+HP	Lubbock
pH	6.67-6.82	6.01-6.09	6.93-7.01
Wet weight (mg)	23.95±4.91	14.70±2.38	30.24±5.58
Dry weight (mg)	2.29±1.92	1.55±0.47	3.11±0.47
Water content (%)	90.46	89.43	89.72
Biomass surface density (mg/cm^2)	12.6±2.58	7.74±1.25	15.92±2.94

Footnote: *S. epidermidis* (ATCC 35983) and *C. albicans* (ATCC 90028) dual-species biofilms were cultivated for 24 hours in 24-well plates in different cultivation media: TSB+HP – Tryptic soy broth supplemented with 10% (v/v) human plasma (HP); RPMI+HP – RPMI 1640 supplemented with 10% (v/v) HP; Lubbock – Bolton broth supplemented with 50% (v/v) HP and 5% (v/v) freeze-thaw lysed sheep red blood cells. Biofilm pH was measured using a pH sensor (InLab Ultra-Micro_ISM, Mettler Toledo). Data are presented as measured pH ranges. For wet weight quantification, biofilms were properly scraped and weighed in Eppendorf tubes using semi-micro balances (SMG 2285, VWR International, LLC Avantor). For dry weight measurements, biofilms in Eppendorf tubes were air-dried (70°C, 24h) and weighed again. The data are expressed as mean ± standard deviation (n=4). The mean percentage of water content (%) was subsequently calculated. The biomass surface density was determined from the wet weight relative to the area of the bottoms of the cultivation plate wells. The data are expressed as mean ± standard deviation (n=4).
